# Fast and simple protein-alignment-guided assembly of orthologous gene families from microbiome sequencing reads

**DOI:** 10.1186/s40168-017-0233-2

**Published:** 2017-01-25

**Authors:** Daniel H. Huson, Rewati Tappu, Adam L Bazinet, Chao Xie, Michael P. Cummings, Kay Nieselt, Rohan Williams

**Affiliations:** 1 Center for Bioinformatics, University of Tübingen, Sand 14, 72076 Tübingen, Germany; 2 Life Sciences Institute, National University of Singapore, 28 Medical Drive, Singapore, 117456 Singapore; 3 Center for Bioinformatics and Computational Biology, University of Maryland, 8314 Paint Branch Drive, College Park, MD 20742 USA; 4 National Biodefense Analysis and Countermeasures Center, 8300 Research Plaza, Fort Detrick, Frederick, MD 21702 USA; 5 Human Longevity Inc., Singapore, Singapore

**Keywords:** Sequence assembly, String graph, Functional analysis, Software

## Abstract

**Background:**

Microbiome sequencing projects typically collect tens of millions of short reads per sample. Depending on the goals of the project, the short reads can either be subjected to direct sequence analysis or be assembled into longer contigs. The assembly of whole genomes from metagenomic sequencing reads is a very difficult problem. However, for some questions, only specific genes of interest need to be assembled. This is then a gene-centric assembly where the goal is to assemble reads into contigs for a family of orthologous genes.

**Methods:**

We present a new method for performing gene-centric assembly, called protein-alignment-guided assembly, and provide an implementation in our metagenome analysis tool MEGAN. Genes are assembled on the fly, based on the alignment of all reads against a protein reference database such as NCBI-nr. Specifically, the user selects a gene family based on a classification such as KEGG and all reads binned to that gene family are assembled.

**Results:**

Using published synthetic community metagenome sequencing reads and a set of 41 gene families, we show that the performance of this approach compares favorably with that of full-featured assemblers and that of a recently published HMM-based gene-centric assembler, both in terms of the number of reference genes detected and of the percentage of reference sequence covered.

**Conclusions:**

Protein-alignment-guided assembly of orthologous gene families complements whole-metagenome assembly in a new and very useful way.

## Background

Functional analysis of microbiome sequencing reads—by which we mean either metagenomic or metatranscriptomic shotgun sequencing reads—usually involves aligning the six-frame translations of all reads against a protein reference database such as NCBI-nr [1], using a high-throughput sequence aligner such as DIAMOND [2]. Each read is then assigned to a functional family, such as a KEGG KO group [3] or InterPro family [4], based on the annotation of the most similar protein reference sequence.

A *gene-centric assembly* for a family of orthologous genes *F* is the assembly of all reads associated with *F*. One approach to this is simply to run an existing assembly tool on the reads. In this paper, we present a new approach to gene-centric assembly that we call *protein-alignment-guided assembly*. We provide an implementation of this approach in the latest release of the metagenomic analysis tool MEGAN Community Edition [5] and will refer to this as the MEGAN assembler. The defining feature of the protein-alignment-guided assembly is that it uses existing protein alignments to detect DNA overlaps between reads. Our implementation of this method is easy to use; it only takes a few mouse clicks to obtain the assembly of any gene family of interest, in contrast to other approaches that require some amount of scripting.

We compare the performance of the MEGAN assembler to that of several standalone assemblers including IDBA-UD [6], Ray [7], and SOAPdenovo [8], and we also compare its performance to that of Xander [9], a gene-centric assembler that employs protein profile HMMs (Hidden Markov Models) rather than sequence alignment to recruit reads. Performance comparisons are based on 41 gene families from a synthetic microbiome community [10].

We use two measures of performance. To assess how well individual gene sequences are *assembled*, we report the percentage of sequence covered by the longest contig that maps to a given reference sequence. To assess how well gene sequences are *detected* for different organisms, we report the number of organisms for which the longest mapped contig covers at least half of the corresponding reference sequence.

All assemblers produce similar numbers of contigs and few, if any, false positive contigs. In our evaluation, we find that the MEGAN assembler performs best in terms of the percentage of reference genes covered and percentage of reference gene sequences detected.

## Methods

The main technical contribution of this paper is the design and implementation of a “protein-alignment-guided” assembly algorithm that is explicitly designed for gene-centric assembly. It is integrated in our metagenome analysis program MEGAN and can be launched and run interactively. The algorithm is based on the concept of a string graph [11] and follows the overlap-layout-consensus paradigm [12]. We use existing protein alignments to infer DNA overlaps and provide a simple path-extraction algorithm to layout reads into contigs. As we only consider perfect overlaps, we obtain a consensus sequence for a contig simply by concatenating reads (accounting for overlaps).

Let *F* be a family of orthologous genes. For example, the KEGG orthology group K03043 represents the DNA-directed RNA polymerase subunit beta, and there are 3216 protein sequences available for this family in the KEGG database. Let *R* denote the set of all reads that are assigned to *F* based on a DIAMOND alignment of all reads against a protein reference database such as NCBI-nr or KEGG.

Our assembly approach is based on an overlap graph. Usually, the set of nodes of an overlap graph is given by the set of reads. However, in our construction, we only use an aligned part of each read *r*, which we call the *aligned core* of *r*. In more detail, we define the aligned core *c*(*r*) of any read *r* to be the segment of the read that is covered by its highest-scoring local alignment to any protein reference sequence in *F*, using the forward or reverse strand of the read, depending on whether the frame of the alignment is positive or negative, respectively.

We build an overlap graph *G* = (*V*,*E*) for *F* as follows. The set of nodes *V* consists of the aligned cores of all reads that have at least one significant alignment to a protein reference sequence. Two such nodes *r* and *s* are connected by a directed edge *e* from *r* to *s* in *E*, if there exists a protein reference sequence *p*∈*F* such that:

1. A suffix of *r* and a prefix of *s* each have a significant alignment with *p*;
2. A suffix of the former alignment overlaps a prefix of the latter;
3. The induced gap-free DNA alignment between *r* and *s* has perfect identity; and
4. The length of the induced DNA alignment exceeds a specified threshold (20 bp, by default).

**Fig. 1:**
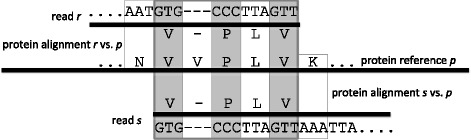
Induced DNA overlap edges. If two reads *r* and *s* both have a protein alignment to the same reference protein *p*, then this defines an overlap edge between the corresponding nodes if the induced DNA alignment has 100% identity. This induced DNA alignment is of length 12, as we ignore any induced gaps

We define the weight ω(*e*) of any such edge *e* to be the length of the induced DNA alignment. See Fig. 1 for an illustration of the overlapping process.

One advantage of using the aligned cores of reads, rather than the complete reads, is that this reduces the need to perform quality trimming of reads, as local alignments should not usually extend into stretches of low-quality sequence. The net effect is that reads are filtered and trimmed, not based on some arbitrary quality valued-associated thresholds as with standard read trimming and filtering procedures, but rather based on the outcome of alignment as protein sequence to reference genes.

We emphasize that overlaps between reads are not only inferred from alignments to just one particular reference sequence (which would be a simple reference-guided assembly), but rather, each read usually participates in overlaps that are induced by alignments to a number of different reference proteins (typically orthologous proteins from different organisms). There is no need to explicitly reconcile these alignments as they only serve to help detect potential DNA overlaps.

The construction of the overlap graph for a given set of reads and associated alignments to protein references is computationally straightforward to implement. We first build a dictionary mapping each reference sequence to the set of reads that align to it. Then, for each reference sequence, we investigate all pairs of overlapping alignments to determine whether to place an overlap edge between the two nodes representing the corresponding aligned cores. Let *k* denote the number of reference sequences on which at least two alignments overlap. In the worst case, all *n* reads align to all *k* references in an overlapping manner and so the number of edges to add to the overlap graph is at most O(*kn* ^2^).

Once the overlap graph has been constructed, the task is to extract contigs from the graph, by first identifying paths through the graph and then computing a consensus sequence from the sequences along those paths. When implementing a sequence assembler under the overlap-layout-consensus paradigm, the layout phase, which consists of determining appropriate paths through the overlap graph that will give rise to contigs, is made difficult by repeat-induced cycles and other artifacts in the overlap graph [12].

Cycles appear only very rarely when assembling the reads of a single gene family. Before processing the graph, we break any directed cycle that exists by deleting the lightest edge in the cycle. Thus, the overlap graph is a directed acyclic graph.

Let *P* = (*r*_1_,*e*_1_,*r*_2_,…,*r*_*n-*1_,e_*n*-1_,*r*_*n*_) be a directed path of edges in the overlap graph *G*, where *e*_*i*_ is the overlap edge between reads *r*_*i*_ and *r*_*i*+1_ for *i* = 1,…,*n* − 1. We define the weight of *P* as ω(*P*) = Σ_*i*_ ω(*e*_*i*_) the total number of overlapping nucleotides along the path.

Our layout algorithm operates by repeating the following steps:

1. Determine a path *P* of maximum weight;
2. Construct a contig *C* by concatenating all read sequences along *P*, accounting for overlaps;
3. Report *C* if the contig exceeds a specified threshold for minimum length and/or minimum average coverage;
4. Remove all nodes and edges of *P* from the graph *G*; and
5. Terminate when no paths remain.

The problem of determining a path of maximum weight in an acyclic-directed graph is solvable in linear time by relaxing vertices in topological order (see [13, p. 661–666]).

We then build a second overlap graph *H* whose set of nodes consists of all contigs assembled from reads. Any two contigs *c* and *d* are connected by a directed edge (*c*,*d*) in *H* if and only if there exists an overlap alignment between a suffix of *c* and a prefix *d* of length ≥20 (by default) and percent identity of at least 98%.

First, we use the graph *H* to identify any contig that is completely contained in a longer one with a percent identity of 98% or more. Such contained contigs are discarded. This addresses the issue that sequencing errors give rise to shorter contigs that differ from longer ones by a small number of mismatches. We then proceed as described above for *G* to assemble the remaining contigs into longer ones, if possible. The number of overlap edges is usually very small, and thus, only a small number of contigs are merged.

The latest release of MEGAN provides an implementation of protein-alignment-guided assembly. The program allows the user to import the result of a BLASTX or DIAMOND alignment of a file of reads against a protein reference database and assigns the reads to nodes in a taxonomy and a number of functional classifications (KEGG, SEED [14], eggNOG [15], or InterPro2GO). When importing a BLASTX file or “meganizing” a DIAMOND file, the user must instruct MEGAN to perform the desired functional classifications by selecting the appropriate check boxes and providing appropriate mapping files that map NCBI accession numbers to functional entities, as described in [5]. In addition, we provide a command-line implementation called gc-assembler for use in a non-interactive setting.

**Fig. 2:**
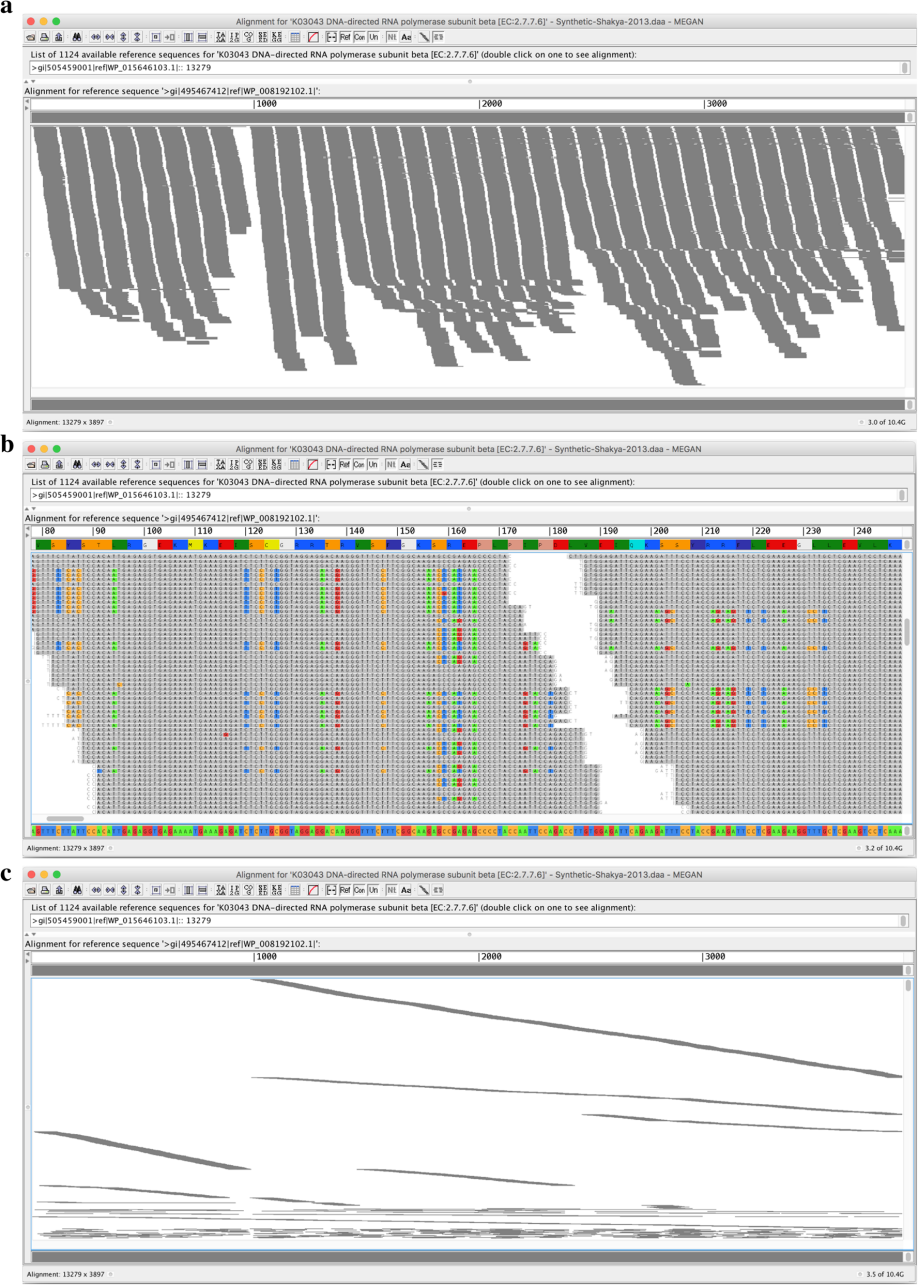
Alignment viewer. **a** Alignment of 13,623 reads against one of the reference sequences representing *bacteria rpoB*, as displayed in MEGAN’s alignment viewer. **b** More detailed view in which nucleotides that do not match the consensus sequence are highlighted in color. **c** Reads are ordered by contig membership and decreasing length of contigs

The MEGAN assembler can be used in a variety of ways. First, the user can select any node(s) in any of the functional classifications to define the gene family or families to assemble. Clicking the “Export Assembly” menu item will then launch the assembler on each of the selected nodes, and the output will be written to one file per gene family in FASTA format. Second, when viewing the alignments of reads against a particular reference sequence in the MEGAN alignment viewer, the user can launch the assembler for the particular reference sequence. In addition, the user can have the alignment viewer layout reads by their membership in contigs. Figure 2a shows the alignment of the reads against a protein reference sequence in the alignment viewer, and Fig. 2b shows the alignment of reads laid out by their membership in contigs.

The main parameters of the assembly algorithm are minOverlapReads, the minimum number of bases that two reads should overlap by; minReads, the minimum number of reads required for a contig; minLength, the minimum length of a contig; minAvCoverage, the minimum average coverage of a contig; minOverlapContigs, the minimum number of bases that two contigs should overlap by to be merged; and minPercentIdentityContigs, the minimum percent identity at which contigs are deemed to overlap or be contained.

To assemble a given gene, MEGAN first needs to extract the associated reads and alignments from the indexed file containing the full set of reads and alignments. This usually takes a couple of minutes. Once this step has been completed, protein-alignment-guided assembly of the reads will take on the order of seconds to minutes (see below).

### Experimental evaluation

We downloaded a dataset of 108 million Illumina reads (54 million per paired-end file) obtained from sequencing a synthetic community containing 48 bacterial species and 16 archaeal species (SRA run SRR606249; [10]). The read length is 101 bp. We aligned these reads against the NCBI-nr database (downloaded February 2015) using DIAMOND (E value cutoff of 0.01), which resulted in more than one billion alignments, involving 87 million reads. We ran the resulting DIAMOND file through our program daa-meganizer, which performs taxonomic and functional assignment of all reads in the dataset based on the alignments found by DIAMOND, and then appends the resulting classifications and indices to the DIAMOND file. This “meganized” DIAMOND file is publicly accessible in MEGAN via MeganServer.

We based our experimental analysis on a set of single-copy phylogenetic marker genes [16] so as to simplify the task of evaluating the performance of the different methods. We also consider some other genes, archaeal and bacterial *rpoB*, *cheA*, *ftsZ*, and *atoB*, to see how the assembly methods perform on other types of genes.

For each gene family in the study, we determined the corresponding KEGG orthology (KO) group and ran the MEGAN assembler on all reads assigned to that family. The assembler was run with the default options of minOverlapReads = 20, minReads = 2, minLength = 200, minOverlapContigs = 20, and minPercentIdentityContigs = 98. In addition, for each KO group, we saved all assigned reads to a file and then assembled them using IDBA-UD, Ray, and SOAPdenovo. All assemblers were run with default options.

To evaluate Xander, we downloaded all associated amino acid and nucleotide KEGG gene sequences for the 41 gene families, aligned the amino acid sequences with MAFFT (using the --auto option; version 7.187 [17, 18]), and built HMMs and configured supporting files according to Xander documentation.

Running Xander using default parameters (min_bits = 50 and min_length = 150) gave rise to small number of contigs per gene family that was much lower than the number of gene family members in the community, resulting in an unacceptable number of false negatives. To address this, we experimented with different parameter settings until Xander produced a number of contigs that is similar to that produced by the other four assemblers. We used the following parameters for Xander: min_bits = 1, min_length = 1, filter_size = 39, min_count = 1, and max_jvm_heap = 64 GB.

Table 1 shows the number of reads assigned to each gene family, as well as the number of reference gene DNA sequences that represent each gene family.

**Table 1:** For each gene family studied, we report the KEGG orthology group, number of reads assigned to that group by DIAMOND, number of reference gene sequences that exist in the synthetic community, and number of reference genes “detected” by each method: MEGAN, IDBA-UD, Ray, SOAPdenovo, and Xander

Gene family	KEGG	Reads	References	MEGAN	IDBA-UD	Ray	SOAP	Xander
Acetyl-CoA C-acetyltransferase	K00626	58,135	64	**31**	16	12	12	23
Archael rpoB1	K03044	17,875	16	7	7	6	3	**9**
Archael rpoB2	K03045	12,025	16	**8**	**8**	6	5	5
Cell division protein	K03531	45,881	48	37	**39**	12	7	12
Bacterial rpoB	K03043	105,212	64	43	16	12	13	**50**
Phenylalanyl-tRNA synthetase alpha subunit	K01889	44,779	64	**57**	56	47	51	48
Phenylalanyl-tRNA synthetase beta subunit	K01890	73,072	64	**53**	50	42	38	35
Phosphoribosylformylglycinamidine cyclo ligase	K01933	31,919	64	58	**59**	46	45	54
Ribonuclease HII	K03470	18,707	64	54	**55**	53	45	48
Ribosomal protein L1	K02863	24,190	64	**57**	49	45	48	53
Ribosomal protein L10	K02864	23,970	64	**58**	48	55	57	55
Ribosomal protein L11	K02867	17,113	64	**60**	50	51	**60**	59
Ribosomal protein L13	K02871	17,642	64	**58**	54	53	57	45
Ribosomal protein L14	K02874	13,435	64	56	42	49	58	**60**
Ribosomal protein L15	K02876	13,087	64	**59**	56	50	55	55
Ribosomal protein L16	K02878	10,058	64	**46**	34	36	44	44
Ribosomal protein L18	K02881	14,856	64	**57**	48	56	**57**	55
Ribosomal protein L2	K02886	29,849	64	**60**	54	46	55	57
Ribosomal protein L22	K02890	15,875	64	**59**	54	55	57	51
Ribosomal protein L24	K02895	11,786	64	**60**	46	56	58	44
Ribosomal protein L25	K02897	12,941	64	41	41	39	**42**	41
Ribosomal protein L29	K02904	4913	64	29	8	33	**34**	8
Ribosomal protein L3	K02906	30,192	64	**59**	47	51	57	51
Ribosomal protein L4	K02926	14,539	64	**44**	41	39	43	**44**
Ribosomal protein L5	K02931	20,533	64	**60**	58	55	59	58
Ribosomal protein L6	K02933	20,645	64	58	41	56	59	**60**
Ribosomal protein S10	K02946	11,327	64	**56**	42	48	**56**	54
Ribosomal protein S11	K02948	10,793	64	47	43	51	52	**56**
Ribosomal protein S12	K02950	14,199	64	**61**	41	48	60	58
Ribosomal protein S13	K02952	13,975	64	59	46	56	**60**	58
Ribosomal protein S15	K02956	10,795	64	54	16	43	**55**	50
Ribosomal protein S17	K02961	10,235	64	58	36	49	44	**60**
Ribosomal protein S19	K02965	12,479	64	**59**	39	51	**59**	58
Ribosomal protein S2	K02967	25,926	64	**61**	46	41	53	48
Ribosomal protein S3	K02982	25,722	64	**59**	46	48	57	57
Ribosomal protein S5	K02988	21,761	64	**59**	55	53	53	56
Ribosomal protein S7	K02992	20,520	64	60	42	54	60	**61**
Ribosomal protein S8	K02994	14,543	64	**62**	57	58	60	57
Ribosomal protein S9	K02996	12,927	64	59	52	52	58	**61**
Signal recognition particle protein	K03110	27,386	64	35	**48**	36	19	46
Two-component system	K03407	47,904	64	**29**	17	15	15	27
Mean absolute deviation				**9.34**	19.73	18.24	15.41	14.17

Best results are shown in bold. Mean absolute deviation between the number of references genes and the number detected by each method is reported as a summary statistic

For a typical gene family with ≈20,000 assigned reads, the MEGAN assembler took approximately 30 s to run, while for other assemblers the time taken was as follows: 55 s (IDBA-UD), 75 s (Ray), and 3 s (SOAPdenovo), on a server with 32 cores. Xander took approximately 1 h (excluding the time required to build the de Bruijn graph) to run on a server with 20 cores. Maximum memory usage was set to 12 GB for the MEGAN assembler and 64 GB for Xander, whereas the other assemblers used 1.5–3 GB of memory.

**Fig. 3:**
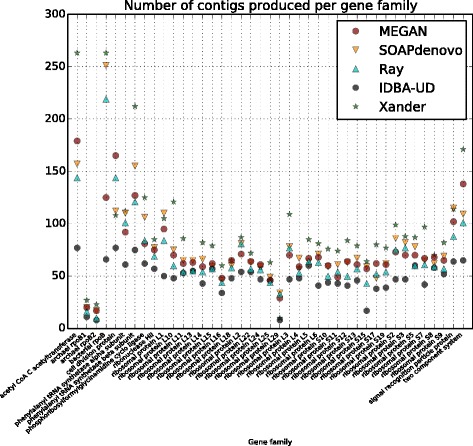
Summary of number of contigs produced. For each gene family along the *x*-axis, we plot the number of contigs of length ≥200 bp produced by each assembler

Using a minimum contig length of 200 bp, all assemblers produced a similar average number of contigs per gene family: 73 (MEGAN), 48 (IDBA-UD), 69 (Ray), 78 (SOAPdenovo), and 93 (Xander). The number of contigs produced per gene family is shown in Fig. 3.

To allow an analysis of the performance of the different methods, we aligned all contigs to all reference genes associated with the synthetic community using BLASTN. In nearly all cases, we found an alignment of at least 98% identity to a reference organism that was part of the synthetic community. In the few remaining cases, a high-identity BLASTX alignment of at least 98% identity to the corresponding protein sequence was found. In nearly all cases, the alignments covered at least 99% of the contig. This indicates that there are only very few, if any, false positive contigs.

**Fig. 4:**
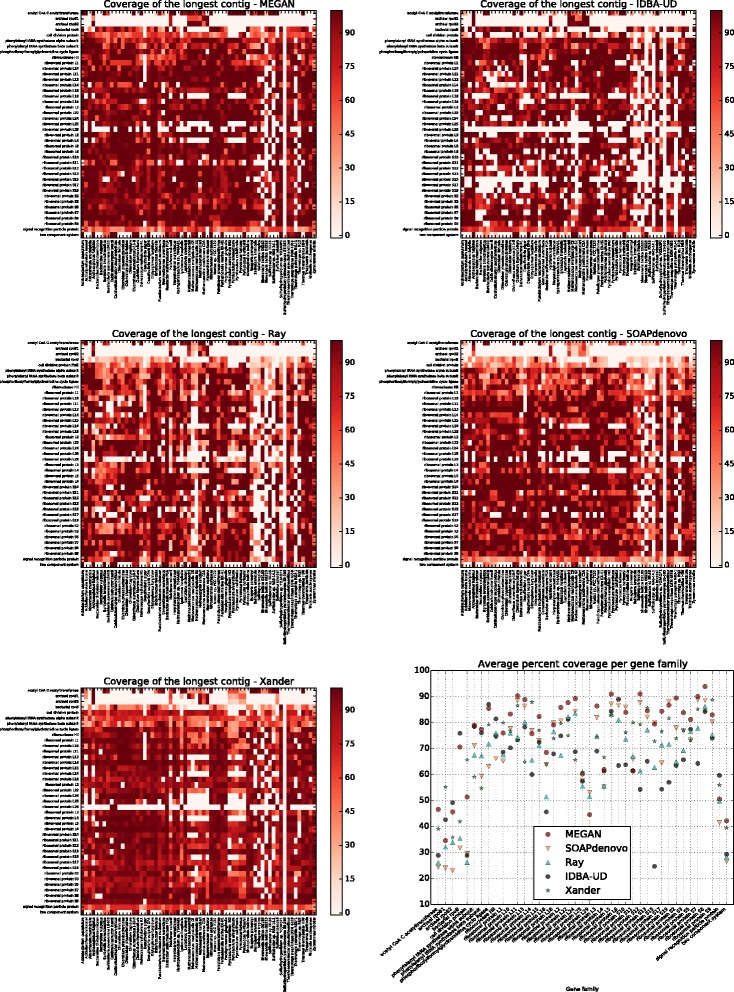
Reference gene coverage heat map. For each assembler, each gene family (rows), and each reference gene sequence associated with a species in the synthetic community (columns), we indicate the percentage of the reference gene covered by the longest contig. We also plot the average percent coverage per gene family for all assemblers

To assess assembly performance, for each assembly, each gene family and each species in the synthetic community that contains a member of the gene family, we determined the percentage of reference gene sequence covered by the longest contig aligned to it (Fig. 4). In this calculation, only matching bases are counted.

**Fig. 5:**
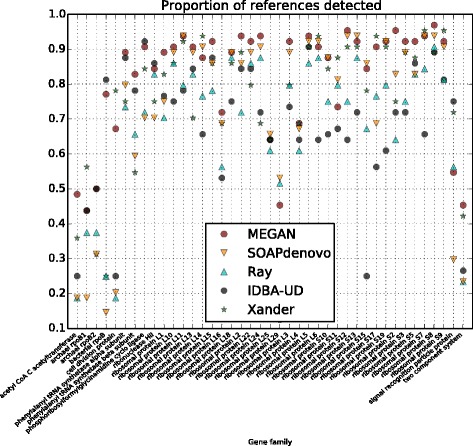
Reference gene coverage summary. For each gene family along the *x*-axis, we plot the number of reference gene sequences detected by each method

To assess detection performance, we consider a reference gene sequence to be successfully *detected* by an assembler if the longest contig covers 50% or more of the sequence, again, counting only matching bases. In Table 1, for each gene and each assembler, we report the number of reference sequences detected (as defined above) and provide a summary of the proportion of reference sequences detected per gene family in Fig. 5, showing that the protein-alignment-based assembler implemented in MEGAN performs best for most genes.

## Results and Conclusions

The work reported on in this paper provides simple and fast access to assemblies of individual gene families from within MEGAN, a popular microbiome sequence analysis tool. Protein-alignment-guided assembly makes use of pre-computed protein alignments to perform gene-centric assembly. Alternative ways of performing gene-centric assembly include running an external assembly tool on the reads assigned to a specific gene family or using an HMM-based framework such as Xander for read recruitment and assembly.

Based on percent coverage by longest contig and number of gene sequences detected, the MEGAN assembler performs best in our experimental study (Figs. 4 and 5). The average percent coverage values over all gene families are 75.4% (MEGAN), 62.4% (IDBA-UD), 64.6% (Ray), 67.8% (SOAPdenovo), and 69.6% (Xander).

Figure 4 indicates that all approaches have difficulties assembling ribosomal protein L29. The reason for this is that members of this gene family are very short, less than 70 aa in many cases, and so the resulting contigs rarely exceed the 200-bp length threshold that we use.

All assembly methods fail on the species *Sulfurihydrogenibium yellowstonense*. In our analysis, only approximately 46,000 (of 108 million) reads are classified as coming from this species and so this species is represented by substantially less reads than the other species in the mock community. The NCBI-nr database contains 1700 reference proteins for this species, and so, the average number of reads assigned to each protein is only 27. In addition, none of these reference sequences is annotated by one of the 41 KOs (KEGG orthology groups) used in this study. So, the failure is due to a combination of low coverage and the absence of any directly corresponding reference sequences.

Note, however, that there are a number of other species (*Bacteroides vulgatus*, *Desulfovibrio piger*, *Gemmatimonas aurantiaca*, and *Salinispora arenicola*) for which there is no directly corresponding reference sequence for any of the 41 KOs, yet for these, the gene-centric assembly appears to work well. In particular, we emphasize that for *Gemmatimonas aurantiaca*, the closest species that has reference proteins for (any and all of) the 41 KOs is *Gemmatirosa kalamazoonesis*, which belongs to a different genus.

In our performance analysis, we assign each contig to at most one organism in the synthetic community. However, in some cases, the same contig aligns equally well to the gene reference sequence of two closely related organisms and as a consequence, the analysis reported in Fig. 4 slightly under-predicts the true performance of all methods. This happens, for example, for ribosomal protein L16 for *Thermotoga sp. RQ2* and *Thermotoga petrophila RKU-1*. We use a threshold of 98% identity to determine whether contigs generated by our assembler are deemed to be containing or overlapping each other. This is to ensure that the number of contigs produced by our assembler is similar to that produced by the other approaches. Increasing the threshold to 99% produces a handful of additional correct gene detections, while roughly doubling the number of contigs.

Gene-centric assembly does not replace the computation of a full assembly of all reads, which remains a challenging problem [19], with some recent advances [20]. DIAMOND alignment of all 108 million reads in the synthetic community [10] against the NCBI-nr database, followed by the gene-centric assembly of all 2834 detected KEGG families using MEGAN, took only one and a half days on a single 32-core server. In contrast, assembly of all 108 million reads from the described synthetic community [10] using Ray-2.3.1 took 6 days on the same server. The resulting assembly contains 52,821 contigs of length ≥200 bp, with a median length of 802 bp, mean length of 3789 bp, and maximum length of 600,408 bp. We estimate that running Xander on all 2834 KEGG families present in the synthetic community will take 10–100 days on a single server with 20 cores.

We emphasize that protein-alignment-guided assembly as currently implemented in MEGAN only constructs contigs that span known protein domains and so interspersed unknown domains will result in contig fragmentation. Here, the application of a full-featured assembler to all protein-alignment-recruited reads and their mates may result in longer contigs that cover some of the unknown domains.

As an all-in-one GUI-based desktop application, MEGAN is especially designed for use by biologists and medical researchers that have limited bioinformatics skills. The built-in assembler now provides such users with simple access to sequence assembly techniques on a gene-by-gene basis.
